# Changes in the Gut Microbiome after Galacto-Oligosaccharide Administration in Loperamide-Induced Constipation

**DOI:** 10.3390/jpm10040161

**Published:** 2020-10-10

**Authors:** Min Guk Kim, Kyungae Jo, Yeok Boo Chang, Hyung Joo Suh, Ki-Bae Hong

**Affiliations:** Department of Integrated Biomedical and Life Science, Graduate School, Korea University, Seoul 02841, Korea; minguk94@gmail.com (M.G.K.); kyungae11@korea.ac.kr (K.J.); oobkoey@gmail.com (Y.B.C.); suh1960@korea.ac.kr (H.J.S.)

**Keywords:** galacto-oligosaccharide, microbiome, constipation, loperamide

## Abstract

Unbalanced dietary habits and the consumption of high protein and instant foods cause an increase in constipation. Here, we evaluated the effects of galacto-oligosaccharide (GOS) on a rat model of loperamide-induced constipation by measuring various biological markers and cecal microbiota. The fecal water content and intestinal transit ratio significantly increased in the GOS-administered (GL and GH) groups than in the control group (*p* < 0.05, *p* < 0.01, and *p* < 0.001, respectively). The length of intestinal mucosa (*p* < 0.05 and *p* < 0.01, respectively) and area of crypt cells were (*p* < 0.01, both) significantly increased in the GOS-administered groups compared to the control group. The distribution of interstitial cells of Cajal, which is related to the intestinal movement, showed a significant increase in GOS-administered groups than in the control group (*p* < 0.01, both). The relative abundance of lactic acid bacteria (LAB), especially *Lactobacillus* and *Lactococcus*, significantly increased in the GL group than in the control group. Furthermore, there was a positive correlation between short chain fatty acids (SCFAs) and the gut microbiota in the GL groups. These results demonstrated that GOS administration effectively alleviates constipation by increasing LAB proliferation in the intestinal microbiota and SCFA production.

## 1. Introduction

In the modern society, the prevalence of constipation is increasing owing to animal-based and instant foods and unbalanced dietary habits [1,2]. Constipation is one of the most common bowel and digestive problems where feces remain in the bowel for a long time and moisture is absorbed by the bowels; it results in infrequent bowel movement, leading to obstructed defecation, prolonged stay in the bathroom, and hardened feces [3]. Constipation is a symptom rather than a disease, the causes determine effective treatment methods such as regular eating habits, laxatives, enema, dietary fiber, and probiotics [4,5].

A primarily fiber-based diet is important for the treatment of constipation, which is best suited for the prevention of constipation. Dietary fiber is a food component that is not digested by intestinal enzymes, but causes absorption of moisture from the colon to make the bowels soft and voluminous [6,7,8] and facilitates bacterial growth in the colon to make the bowels bigger. Furthermore, insoluble fibers are fermented by the colon bacteria, and the resulting metabolites help relieve constipation [7].

Prebiotics have recently begun to replace probiotics because of increased resistance to digestive barriers, reduced costs, reduced risks, and easier inclusion into the diet. In addition to prebiotics such as inulin, fructo-oligosaccharide (FOS), and galacto-oligosaccharide (GOS), isomalto-oligosaccharide, xylo-oligosaccharides, resistant starch, and soybean oligosaccharides are used as prebiotics [9].

GOS is a typical oligosaccharide represented by Gal-(Glc)n-Glc. It reportedly facilitates the proliferation of intestinal beneficial bacteria, namely *Bifidobacteria* [10]. GOS has various physiological functions such as improving intestinal and liver functions, strengthening blood pressure, anti-cancer activity, and prevention of osteoporosis, as indicated by clinical tests. On the contrary, the side effect of taking excessive GOS is only temporary bloating; thus, GOS is considered to be very safe [11,12].

Previous studies focused on manufacturing GOS and investigating various physiological activities. In this study, we aimed to investigate the intestinal health, physiological function, and microbiome composition following GOS administration. The constipation mitigation function of GOS and its effect on short chain fatty acids (SCFAs) and the gut microbiome were assessed in a rat model of loperamide-induced constipation.

## 2. Materials and Methods

### 2.1. Animals and Reagents

Six-week-old male Sprague-Dawley rats (n = 30; average body weight: 160–180 g) used in the experiment were purchased from Orient Bio Inc. (Seongnam, Korea). The rats were housed in a specific pathogen-free room at 24 ± 2 °C with 50 ± 5% relative humidity and lighting (12 h day/night cycles). The rats were given free access to tap water and the standard maintenance diet (Purina rodent chow) during the experiment and acclimated for 1 week before use. All experiments were conducted in accordance with the experimental regulation of Institutional Animal Care and Use Committee at Korea University (KUIACUC-2019-91). GOS (the degree of polymerization (DP) ≥ 2: 21.00 ± 5.66; DP ≥ 3: 31.00 ± 7.07; DP ≥ 4: 20.50 ± 6.36) was obtained from Neo Crema Co., Ltd. (Seoul, Korea). Loperamide (L4762) and phenolphthalein (105945) were purchased from Sigma-Aldrich (St. Louis, MO, USA).

### 2.2. Experimental Design

The rats were randomly divided into five groups (n = 6/group): Normal (non-constipated group), control (loperamide-induced constipation group), positive control (PC: 70 mg/kg phenolphthalein-administrated group after loperamide-induced constipation), low dose of GOS-administrated group (GL: 100 mg/kg GOS-administrated group after loperamide-induced constipation), and high dose of GOS-administrated group (GH: 200 mg/kg GOS-administrated group after loperamide-induced constipation). Constipation was induced by oral administration of loperamide (3 mg/kg) for 7 days in rats in all groups except those in the normal group. After induction of constipation, the treatments corresponding to each group were orally administered for 16 days; rats in the normal group were orally administered physiological saline. Food and drinking water intake and body weight were measured daily. 

### 2.3. Measurement of Fecal Parameters

The fecal number, weight, and water content were measured by dividing the period of loperamide administration and the period in which constipation was induced after loperamide administration. The feces were collected every 2 days during the loperamide administration period and a total of four times during the period of loperamide-induced constipation. To measure the fecal weight, the water content of the feces was dried in a 70 °C dry oven for 24 h and the weight difference before and after drying was calculated.

### 2.4. Intestinal Transit Ratio

The intestinal transit ratio was measured by a modified method described by Baik et al. [13]. Briefly, the experimental animals were orally administered with 1 mL of 8% charcoal and sacrificed after 20 min to remove the gastrointestinal tract. The distance traveled was expressed as a percentage of the total length of the intestine from the gastro-pyloric junction to the ileocecal junction.

T (%) = B/A × 100 T: The intestinal tract ratio of charcoal A: Total length of intestinal tract B: Moving distance of the most distal end portion of the charcoal

### 2.5. Analysis of SCFAs

SCFAs in the cecum were determined by gas chromatography (GC; YL-6100 GC system, Young Lin Co., Anayang, Korea) equipped with a capillary column DB-FFAP 123-3253 (50 m × 0.32 mm × 0.50 μM), flame ionization detector, and an autosampler (HT 300, Young Lin Co.). Methanol (80%, 5 mL) was added to 0.5 g of cecal contents and mixed by vortexing for 5 min. The filtrate obtained by filtration using a 0.45-μm Millipore filter (Millipore, Burlington, MA, USA) and after centrifugation (8000× *g*, 20 min, 4 °C) was used for analysis. One microliter of the sample was used for GC. GC inlet and detector temperatures were 200 and 240 °C, respectively, and the analysis conditions were based on the method used by Demigne and Remesy [14].

### 2.6. Histological Observation

The transverse colon obtained from the SD rats was fixed with 10% formalin for 12 h, embedded in paraffin, sectioned into 5-μm-thick slices, and stained with hematoxylin and eosin (H&E, Sigma-Aldrich). Images were with a stereoscopic microscope at 63× magnification (Axio Zoom v.16; Carl Zeiss, Göttingen, Germany) and morphological features of intestinal muscoa cells were evaluated via ImageJ software (National Institutes of Health, public domain). In addition, fixed tissue fragments were embedded in paraffin and continuously cut into 5-μm-thick sections. The sectioned fragments were subjected to Alcian blue staining (pH 2.5) and neutral red counterstaining. Alcian blue-positive intestinal mucosal cells were observed with an optical microscope and the Leica Application Suite software (Leica Microsystems, Switzerland) was used for morphological observation. For analysis of Alcian blue intensity (mucins staining), 10 random cryptic cells from at least five fields of view were analyzed per sample using the MATLAB software.

### 2.7. Immunohistochemistry (IHC) Staining of Interstitial Cells of Cajal (ICC)

ICC are cells located within the soft muscle layer of the intestine; they assist in the peristaltic movement of the intestine. To visualize these cells, the intestinal tissues were paraffin-embedded as described in Section 2.6, sectioned into 5-μm-thick slices, and rehydrated with decreasing ethanol concentrations (100, 90, 80, and 70%). After heating the glass sides with the tissue at 97 °C for 20 min for antigen retrieval, pre-antibody blocking was performed to prevent staining of cells other than ICC. For the primary antibody, the ABC kit (Santa Cruz; SC-168, Dallas, TX, USA) was used and diluted at 1:500 to react at 4 °C for 1 day. After the reaction, the tissue was treated with an antibody enhancer and incubated for 10 min. After treatment with Chromogen, the tissue was washed for 1–2 min with running water, and after 5 min, counterstained with hematoxylin, washed with PBS and running tap water, and then mounted. The stained ICC was observed with an optical microscope (MM-400, Nikon, Tokyo, Japan) and analyzed using the MATLAB program.

### 2.8. Analysis of Pro-Inflammatory Cytokine Levels

For the cytokine assay, ELISA kits for rat interleukin 1 beta (IL-1β), IL-6, and tumor necrosis factor-alpha (TNF-α) were purchased from Koma Biotech (Seoul, Korea). Centrifuge samples at 2000× *g* for 15 min at 4 °C to remove clots, and serum was used to measure the cytokine level, according to the manufacturer’s instructions. The samples or standards were incubated on a 96-well plate for 2 h at room temperature. After washing, the detection antibodies and conjugates were sequentially used. Color alterations were read using a NanoQuant pro200 instrument (Tecan, Mannedorf, Switzerland) at 450 nm.

### 2.9. Analysis of 16S rRNA Gene Sequence and Microbial Community of Cecal Microbiota

For extraction of metagenomic DNA of cecum microorganisms, an UltraClean^®^ Fecal DNA Isolation Kit (MO BIO Laboratories, Carlsbad, CA, USA) was used [15]. Using pyrosequencing technology, a high-throughput analysis method for the 16S rRNA gene, the microbiota in cecum was analyzed. Microbial analysis was performed using Illumina 16S V3~V4 Amplicon (Illumina, San Diego, CA, USA) and a hypervariable region of the 16S rRNA gene as a target. Among the DNA sequences of each sample analyzed by Illumina MiSeq, sequences and barcodes of 300 bp or less were removed during the quality check. Furthermore, after low-quality and non-target sequences were removed using MOTHUR, a data analysis program [16], representative sequences were finally obtained and used for analysis. Operational taxonomic units (OTUs) with a 97.0% identity threshold were analyzed using the CD-HIT program. Taxonomic ranking and classification were classified according to the cut-off criteria [(species(x ≥ 97%), genus (97% > x ≥ 94%), family (94% > x ≥ 90%), order (90% > x ≥ 85%), class (85% > x ≥ 80%), and phylum (80% > x ≥ 75%)] using the EzTaxon-e database [17].

The diversity and abundance of microorganisms present in the feces were measured based on > 97% homology using MOTHUR (v. 1.32.1). The significance analysis of microbial diversity (Shannon index) and abundance (ACE index) between groups was performed using the Kruskal-Wallis test method (*p* < 0.05). Statistical analysis was performed using the statistical package for social science (SPSS, SPSS Inc., Chicago, IL, USA) software; the significance analysis for the relative ratio between each group was conducted using one-way analysis of variance (ANOVA) and the Dunnett’s test for post-hoc analysis.

### 2.10. Statistical Analysis

The statistical analysis of all results was processed using the SPSS software (version 25.0), and the significance test between each experimental group was conducted using ANOVA and post-hoc Tukey’s test.

## 3. Results

### 3.1. Fecal Water Content and Number and Weight of Fecal Pellets

**Figure 1:**
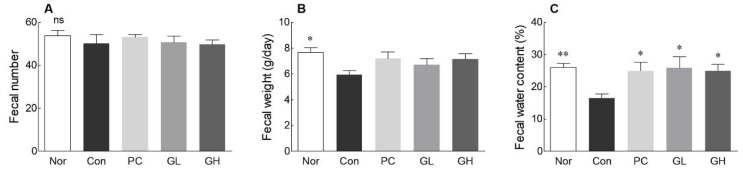
Effects of galacto-oligosaccharide (GOS) administration on fecal number (**A**), fecal weight (**B**), and fecal water content (**C**) in loperamide-induced constipated rats. Data are represented as the mean ± standard error of the mean, and different symbols indicate significance at ** *p* < 0.01 and * *p* < 0.05 vs. control group. Nor: Normal group; Con: Loperamide-treated group; PC: 70 mg/kg phenolphthalein-administrated group after loperamide-induced constipation; GL: Low dose (100 mg/kg) of GOS-administrated group after loperamide-induced constipation; GH: High dose (200 mg/kg) of GOS-administrated group after loperamide-induced constipation; ns: Not significant.

After constipation, the fecal weight and water content of the normal group rats tended to be significantly higher than those of the control group rats (*p* < 0.05, Figure 1). On the contrary, there was an insignificant difference in the fecal number between the normal and control groups. Furthermore, the fecal water content was the lowest in the control group (16.5%). The fecal moisture content in the treatment groups (PC, GL, and GH) was significantly higher than that in the control group. There was no significant difference in the fecal moisture content between the treatment groups (PC, GL, and GH) and the normal group.

### 3.2. Effects of GOS on the Intestinal Transit Ratio

**Figure 2:**
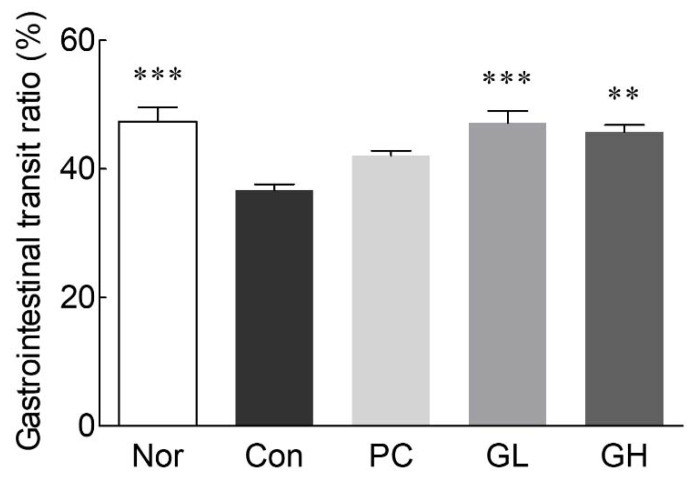
Effects of galacto-oligosaccharide (GOS) administration on gastrointestinal transit ratio in loperamide-induced constipated rats. Data are represented as the mean ± standard error of the mean, and different symbols indicate significance at ** *p* < 0.01 and *** *p* < 0.001 vs. control group. Nor: Normal group; Con: Loperamide-treated group; PC: 70 mg/kg phenolphthalein-administrated group after loperamide-induced constipation; GL: Low dose (100 mg/kg) of GOS-administrated group after loperamide-induced constipation; GH: High dose (200 mg/kg) of GOS-administrated group after loperamide-induced constipation.

Figure 2 shows the intestinal transit ratio. The intestinal transit ratio in the control group (36.7%) was significantly lower than that in the normal group (47.4%, *p* < 0.001). The oral administration of a low and high dose of GOS tended to increase the intestinal transit ratio, and there were significant differences (*p* < 0.001 and *p* < 0.01, respectively) when compared to that of the control group. However, there was no significant difference in the intestinal transit ratio between the normal and GOS-administered groups (GL and GH).

### 3.3. Production of SCFAs

**Figure 3:**
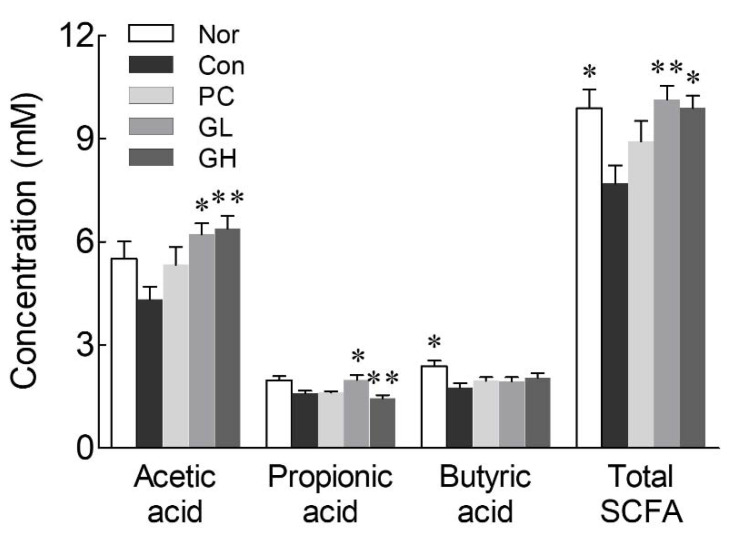
Effects of galacto-oligosaccharide (GOS) administration on short chain fatty acids in loperamide-induced constipated rats. Data are represented as the mean ± standard error of the mean, and different symbols indicate significance at ** *p* < 0.01 and * *p* < 0.05 vs. control group. Nor: Normal group; Con: Loperamide-treated group; PC: 70 mg/kg phenolphthalein-administrated group after loperamide-induced constipation; GL: Low dose (100 mg/kg) of GOS-administrated group after loperamide-induced constipation; GH: High dose (200 mg/kg) of GOS-administrated group after loperamide-induced constipation; SCFA: Short chain fatty acid.

The amount of SCFAs, which promote intestinal health, in cecum was analyzed by GC to evaluate the effect of GOS on SCFA production (Figure 3). GOS administration (100 and 200 mg/kg) significantly increased the concentration of total SCFAs compared to that of the control group. In particular, the GL and GH groups exhibited a dose-dependent increase in acetic acid content, which was higher than that of the normal and control groups (Figure 3). There was no significant difference in the total SCFA amount between the normal and the GOS-administered groups. 

### 3.4. Histological Observations and IHC Staining of ICC

**Figure 4:**
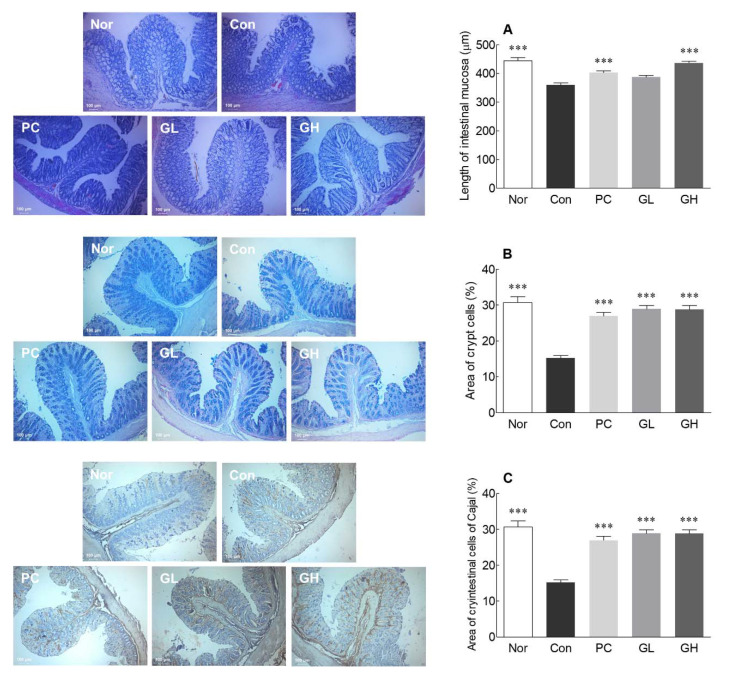
Effects of galacto-oligosaccharide (GOS) administration on length of intestinal mucosa (**A**), area of crypt cells (**B**), and area of interstitial cells of Cajal (ICC) (**C**) in loperamide-induced constipated rats. Data are represented as the mean ± standard error of the mean, and different symbols indicate significance at *** *p* < 0.001 vs. control group. Nor: Normal group; Con: Loperamide-treated group; PC: 70 mg/kg phenolphthalein-administrated group after loperamide-induced constipation; GL: Low dose (100 mg/kg) of GOS-administrated group after loperamide-induced constipation; GH: High dose (200 mg/kg) of GOS-administrated group after loperamide-induced constipation.

Histological data are widely used to demonstrate significant changes in the intestines of constipated animals. For histological analysis, H&E and Alcian blue staining were performed to analyze the thickness of the mesentery membrane and the distribution of crypt cells involved in mucin production. It is known that when constipation is induced by loperamide, the thickness of the large intestinal mucosa decreases, delaying the movement of the contents in the large intestine [18]. The thickness of the intestinal mucosa, as observed through H&E staining, was significantly lower in the control group rats than in the normal group rats (Figure 4A). GOS administration (low and high dose) significantly increased the thickness of the intestinal mucosa compared to that in the control group rats (*p* < 0.05). The thickness of the intestinal mucosa was at a similar level in rats in the GL and PC groups, and the GH and normal groups. The thickness of the intestinal mucosa was recovered after GOS administration.

The distribution of the crypt cell, a mucous secretory cell, was visualized using Alcian blue staining and found significant reduction in the PC group rats compared to the normal group rats (Figure 4B). On the contrary, the PC group had significantly greater distribution of crypt cells than that in the control group rats (Figure 4B). It was also confirmed that crypt cell distribution was significantly increased in the GL and GH group rats compared to that in the control group rats. It is suspected that GOS treatment increases the number of mucus-secreting cells to restore mucus secretion, thereby improving constipation. The thickness of the epithelial tissue layer and the density of crypt cell containing mucin in distal colon were reduced by loperamide treatment but recovered by GOS administration.

IHC staining was used to assess the distribution of ICC in the intestine. The control group rats had significantly lower, while PC group rats had significantly greater, distribution of ICC than the normal group rats (Figure 4C). ICC distribution was significantly increased in the GL and GH group rats compared to that in the control group rats. GOS administration was thought to increase the distribution of ICC that was decreased due to constipation and aid intestinal peristalsis.

### 3.5. Effects of GOS on Levels of Pro-Inflammatory Cytokines

**Figure 5:**
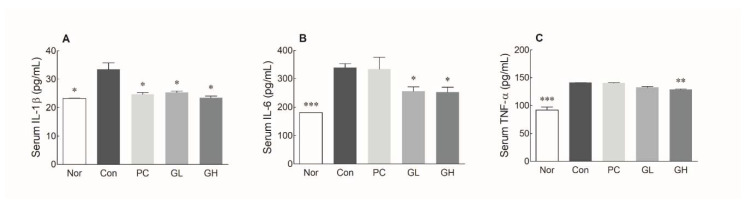
Effects of galacto-oligosaccharide (GOS) administration on the levels of serum IL-1 beta (**A**), IL-6 (**B**), and tumor necrosis factor-alpha (**C**) in loperamide-induced constipated rats. Data are represented as the mean ± standard error of the mean, and different symbols indicate significance at *p < 0.05, **p < 0.01 and *** *p* < 0.001 vs. control group. Nor: Normal group; Con: Loperamide-treated group; PC: 70 mg/kg phenolphthalein-administrated group after loperamide-induced constipation; GL: Low dose (100 mg/kg) of GOS-administrated group after loperamide-induced constipation; GH: High dose (200 mg/kg) of GOS-administrated group after loperamide-induced constipation.

To investigate the effects of GOS on the inflammatory response, the levels of pro-inflammatory cytokines including IL-1β, IL-6, and TNF-α were measured in the serum using ELISA analysis. The serum levels of IL-1β, IL-6, and TNF-α were significant differences between the Nor and Con groups (Figure 5A: *p* < 0.05; Figure 5B,C: *p* < 0.001). Phenolphthalein administration significantly decreased the level of IL-1β compared to the Con group (Figure 5A: *p* < 0.05), but there was no significant difference in the levels of the other two cytokines. The GOS-administered groups (GL and GH) showed decreased IL-1β and IL-6 levels in the serum compared to the Con group (Figure 5A,C: *p* < 0.05). In addition, in the high dose of GOS-administrated group, significantly reduced TNF-α level was confirmed when compared to the Con group (Figure 5C: *p* < 0.01). These data suggest that GOS administration can inhibit the levels of pro-inflammatory cytokines such as IL-1β, IL-6, and TNF-α in the serum of a loperamide-induced constipated rat. In addition, the results of the transcript levels in the colon showed similar results to serum cytokine levels (Supplementary Figure S1).

### 3.6. Effects of GOS on Cecal Microbiota Diversity and Taxonomic Composition of Cecal Microbiota at the Phylum Level

**Figure 6:**
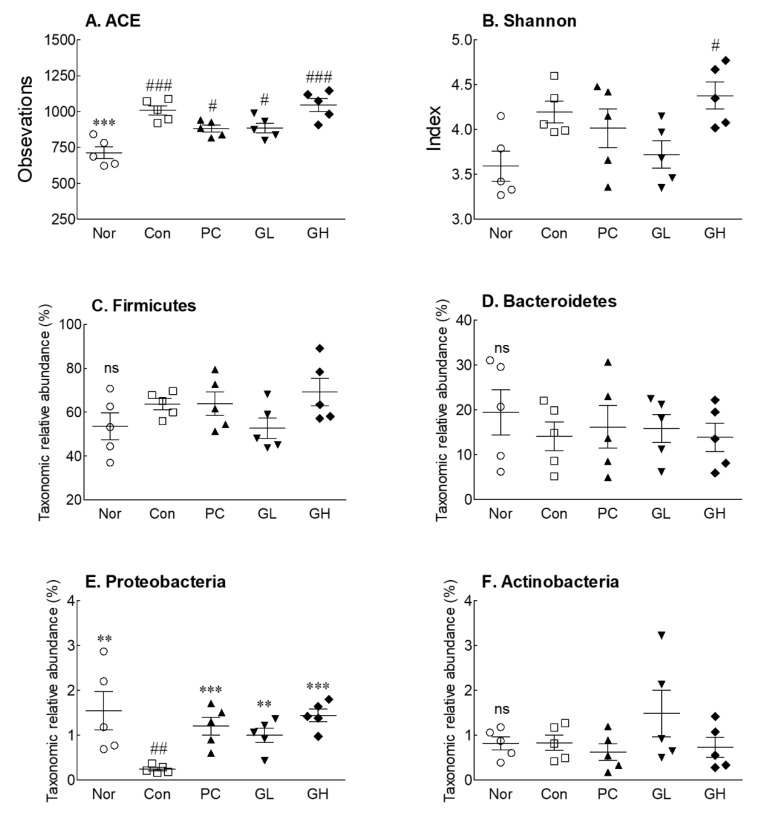
Alpha diversity index (ACE index), beta diversity index (Shannon index), and relative abundance of main phyla in cecal microbiota of loperamide-induced constipated rats. Data are represented as the mean ± standard error of the mean, and different symbols indicate significance at ** *p* < 0.01 and *** *p* < 0.001 vs. control group and ^#^ *p* < 0.05, ^##^ *p* < 0.01 and ^###^ *p* < 0.001 vs. normal group. Nor: Normal group; Con: Loperamide-treated group; PC: 70 mg/kg phenolphthalein-administrated group after loperamide-induced constipation; GL: Low dose (100 kg/mg) of galacto-oligosaccharide (GOS)-administrated group after loperamide-induced constipation; GH: High dose (200 mg/kg) of GOS-administrated group after loperamide-induced constipation; ns: Not significant.

To analyze the effect of GOS on cecal microbiota, metagenomic alpha analysis of the V3-V4 region of the 16S rRNA gene sequence was performed. Figure 6 shows the diversity index for community richness (abundance-based coverage estimator, ACE) and the community diversity index (Shannon) measured at the OTU level.

There was a significant difference in the ACE index, the community richness index of cecal microbiota, between the loperamide-induced constipated groups (Control, PC, GL, and GH) and the normal group, but there were no significant differences between the loperamide-induced constipated groups (Figure 6A). The Shannon index, which is an indicator of diversity of the microbiota, was significantly different between the GH and normal groups, but not the other groups (Figure 6B). Loperamide treatment had an effect on richness and diversity; it seemed to have a greater impact on the richness in particular.

In the composition of the cecum microbial group, changes in the microbiota, ranging from phylum to genus, were observed (Supplementary Figure S2). Firmicutes and Bacteroidetes were predominant at the phylum level, but no significant differences were observed between the groups (Figure 6C,D). In the normal and control groups, Bacteroidetes tended to increase, while in the GL and GH groups, they tended to decrease, compared to those in the control group (Figure 6D). The normal, GL, and GH groups showed significant differences in Proteobacteria when compared to those in the control group (Figure 6E). Furthermore, there were no significant differences in the relative abundance level of Actinobacteria between all groups (Figure 6F).

### 3.7. Effects of GOS on Taxonomic Composition of Cecal Microbiota at the Genus Level

**Figure 7:**
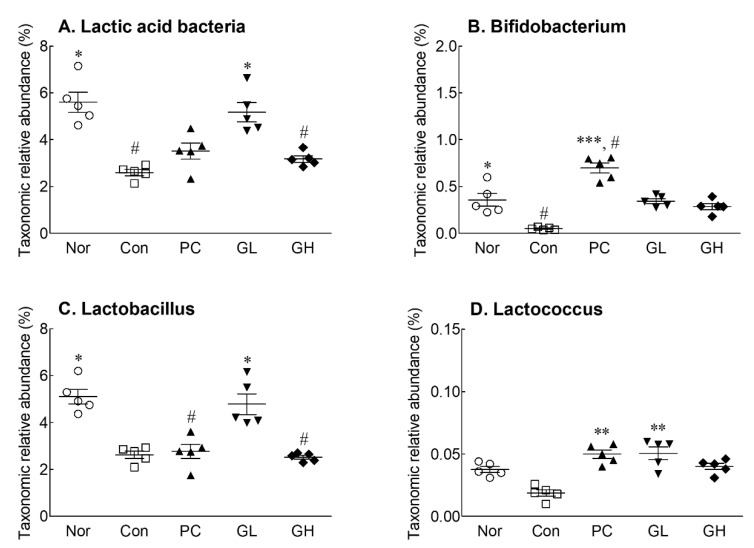
Relative abundance of main lactic acid bacteria in cecal microbiota of loperamide-induced constipated rats. Data are represented as the mean ± standard error of the mean, and different symbols indicate significance at * *p* < 0.05, ** *p* < 0.01, *** *p* < 0.001 vs. control group and ^#^ *p* < 0.05 vs. normal group. Nor: Normal group; Con: Loperamide-treated group; PC: 70 mg/kg phenolphthalein-administrated group after loperamide-induced constipation; GL: Low dose (100 mg/kg) of galacto-oligosaccharide (GOS)-administrated group after loperamide-induced constipation; GH: High dose (200 mg/kg) of GOS-administrated group after loperamide-induced constipation.

Changes in the composition of the cecal microbial group were observed at the genus level, especially for changes in lactic acid bacteria (LAB) (Figure 7). The relative abundances of LAB were decreased during loperamide-induced constipation (Figure 7A). In particular, the relative abundances of *Lactobacillus*, which accounts for a large proportion of LAB, and *Bifidobacterium* were significantly decreased (Figure 7B,C). The relative abundance of LAB was significantly increased in the GL group compared to that in the control group, especially the relative abundance of *Lactobacillus* and *Lactococcus* (Figure 7).

### 3.8. Correlation between Intestinal Microbiota and Biological Indexes

**Figure 8:**
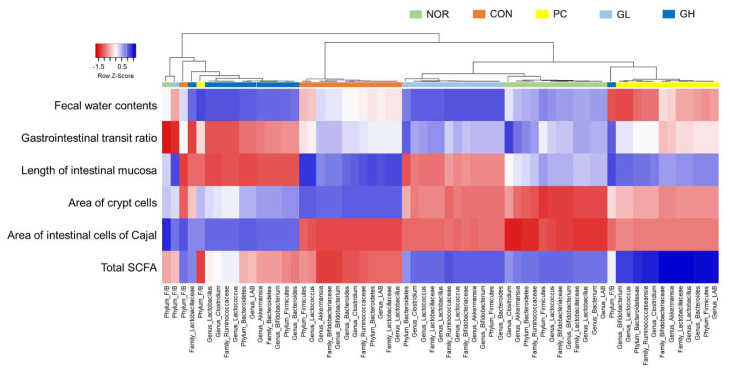
Pearson correlation analysis of the cecal bacterial communities and biological indexes. Nor: Normal group; Con: Loperamide-treated group; PC: 70 mg/kg phenolphthalein-administrated group at 70 mg/kg after loperamide-induced constipation; GL: Low dose (100 mg/kg) of galacto-oligosaccharide (GOS)-administrated group after loperamide-induced constipation; GH: High dose (200 mg/kg) of GOS-administrated group after loperamide-induced constipation; SCFA: Short chain fatty acid.

The correlation between biological indexes and intestinal microbiota was performed using the Pearson correlation analysis. As shown in Figure 8, normal, PC, and GL groups showed similar correlations between biological indexes and microbiota, but these groups showed different correlations than the control and GH groups. In particular, there was a positive correlation between SCFAs and microbiota in the PC, normal, and GL groups, whereas there was a negative correlation with the corresponding strains in the control and GH groups. 

A correlation was found with intestinal microbiota in PC, normal, and GL groups. The normal, GH, and GL groups that showed significant differences compared to the control group regarding SCFAs showed a positive correlation with Bacteroidetes and LAB, whereas the control group showed a negative correlation. For the remaining indicators, it was difficult to find a correlation with microbiota. Vandeputte et al. [19] described a positive correlation between the Ruminoclassae-Bacteroidetes enterotype and constipation improvement among microbiota. Changes in SCFA production between the groups also show a positive correlation with these strains (Figure 8). 

The number of samples did not seem to be sufficient to analyze the correlation between the gut microbiota and the biomarker. The analysis of a large number of samples is required for correlation analysis, and we intend to proceed with this in the future.

## 4. Discussion

The delay in colorectal movement caused by loperamide leads to a decrease in the frequency of feces and an increase in bowel contraction, resulting in constipation [20]. Loperamide-induced suppression of intestinal fluid secretion and colon peristalsis [21] extend the fecal secretion time and delay the intestinal passage [22]. Therefore, loperamide-induced constipation is considered a model of spastic constipation [23]. As shown in Figure 1, GOS oral administration tended to increase the fecal water content to relieve loperamide-induced constipation. 

The intestinal transit ratio is important for the diagnosis of constipation because it reflects the overall intestinal motility [24]. Compared to that in the control group, an increase in the intestinal transit ratio following GOS administration suggests that it may improve intestinal motility, which is associated with colonic movement. It has been reported that such an increase in bowel movement can be expected to relieve constipation [25]. Consistently, increased intestinal movement during loperamide-induced constipation by oral GOS administration showed the anti-constipation effect of GOS. Moreover, oral GOS administration increased defecation frequency per week in the results of double-blind randomized controlled trial, and considerable evidence indicates that consumption of GOS has various health-related effects such as gut function and the growth of beneficial bacteria [26].

The intestinal microorganism ferment dietary fiber produces SCFAs, mainly acetic acid, propionic acid, and butyric acid. In the intestine, SCFAs not only act as nutrients for intestinal epithelial cells but also serve to regulate intestinal pH, cell proliferation and differentiation, and gene expression [27]. Acidification of the intestinal environment by the production of SCFAs decreases the solubility of bile acids, increases mineral absorption, and reduces ammonia absorption and the number of harmful bacteria that inhibit growth under acidic conditions [28]. SCFAs lower the incidence of irritable bowel syndrome and inflammatory bowel disease and reduce the risk of cancer, cardiovascular disease, and obesity [27]. In particular, SCFAs promote the regeneration of the damaged colon and protect the large intestine, thereby enhancing colon function [29]. Indigestible oligosaccharides are known to function as SCFA-producing agents in the large intestine. Potential substrates involved in SCFA production include FOS, oligofructose, GOS, and xylo-oligosaccharides [30,31,32,33]; these oligosaccharides are reportedly fermented by microorganisms present in the large intestine to make metabolites such as SCFAs, which are absorbed by the host to maintain intestinal health [30]. Therefore, increased production of SCFAs by GOS consumption will help improve the intestinal function. 

The colon mucus is covered with a layer of mucus gel that protects the epithelium from mechanical damage and chemical stimuli [34]. Mucin is a major component of mucus and has physical and chemical properties [35]. Loperamide reduces colon mucus, mucus layer thickness in the distal colon, and the amount of mucin released [36]. The gastrointestinal tract has neurological and extraneural functions, among which the phasic contraction is caused by spontaneous electrical activity called a slow wave [37]. The slow wave occurs in the ICC, which is electrically connected to the colon smooth muscle through a gap connection. It is known that the slowing constipation reduces the number of ICC in the colon smooth muscle [38]. ICC are found between the nerve endings and smooth muscle cells in the gastrointestinal tract and are known to have a close relationship with constipation [39]. ICC have generally been reported as pacemaker cells and neuromuscular transport mediators for gastrointestinal activity [40]. In patients with constipation, the distribution of ICC in the colon is significantly lower than that in non-constipated subjects [41]. A decrease in ICC distribution causes a lack of slow wave activity, affecting the intestinal contraction response, thereby delaying fecal passage. As GOS improves the distribution of ICC, it may have an anti-constipation effect.

Disturbances in bowel function is known to lead to disordered immunity and to decreased resistance to pathogenic bacteria in the constipated patients [42]. Dietary prebiotic consumption is known to stimulate the immune system through increasing population of beneficial bacteria or probiotics such as lactic acid bacteria and *Bifidobacteria*. Dietary oligosaccharides show immune-modulating effects through alteration of the intestinal microbiota or the microbiota-independent immune manner [43]. GOS has been studied widely for altered phagocytosis, natural killer cell activity, and anti-inflammatory cytokine production, along with a modulation of the fecal microbiota profile [44,45]. In addition, we observed a significant reduction in the production of pro-inflammatory cytokines (IL-1β, IL-6, and TNF-α) through oral administration of GOS in loperamide-induced constipated rats.

Kashyap et al. [46] indicated that slow passage times and constipation induced by pharmacological agents such as loperamide cause changes in the intestinal microbial community. As such, the change in richness and diversity between normal and other groups is presumed to be due to loperamide. Intestinal disease is closely related to intestinal microbiota, and regulation of intestinal microbiota is known to affect intestinal disease [47,48]. Loperamide affects the abundance and diversity of the microbial community, and GOS influences the same. It is known that the relative abundance of Firmicutes and Actinobacteria decreases when constipation is induced [49], but only a decrease in Actinobacteria was observed in this study. In addition, the study findings were consistent with the notion that the relative abundance of Bacteroidetes increases when constipation is induced. However, the relative abundance of Firmicutes and Proteobacteria can be restored with tagatose [50] or Shenzhu Capsule [51] treatment. Moreover, when GOS was administered, the abundance of Proteobacteria, rather than Firmicutes, was restored.

Loperamide changes the intestinal microbiota, leading to severe destruction of the intestinal microbiome. In particular, a decrease in *Lactobacillus, Bifidobacterium, Roseburia, Anaerotruncus,* and *Lachnospiraceae* was found in the loperamide-treated group and an increase in the PC phenolphthalein- and mulberry-treated group [50]. LAB seem to act as an important factor in constipation; thus, an increase in LAB, among the gut microbiota, may increase the possibility of improving constipation. Eor et al. [52] demonstrated the relative increase in the *Enterobacteriaceae* family and a decrease in the *Bifidobacterium* and *Lactobacillus* genus, Clostridium group (Cluster IV), and *Faecalibacterium prausnitzii* in the loperamide-administered constipation group. In our previous study [53], we confirmed that GOS is involved in the proliferation of *Lactobacillus acidophilus*, *L. casei*, *Bifidobacterium bifidum*, and *B. longum* in an in vitro culture. In addition, when 1% GOS was administered to rats, the growth of *B. bifidum* and *B. longum* in the intestine increased rapidly for up to 12 h, and then slowly increased. High doses of prebiotics (FOS and GOS oral intake at 16 g/day) reported an increase in *Bifidobacteria*, but a decrease in butyrate producing bacteria such as *Phascolarctobacterium* and *Ruminococcus* was reported [54]. GOS administration improved lactose digestion and tolerance by increasing the relative abundance of lactose-fermenting *Bifidobacterium*, *Faecalibacterium*, and *Lactobacillus* in lactose-intolerant individuals, and showed a clear bifidogenic effect on the resident gut microbiota of obese adults [55,56]. In addition to the improvement of the intestinal flora by GOS, some prebiotics including GOS may have anti-adhesive activity and have been reported to inhibit the adherence of pathogenic bacteria to the surface of host epithelial cells [57]. Therefore, GOS consumption seems to contribute to the relief of constipation by improving the changes in the microbiota, particularly restoring the abundance of LAB, caused by constipation.

## 5. Conclusions

The findings of this study suggest that oral GOS administration leads to increased moisture content of feces, maintenance of integrity of intestinal mucosal cells, crypt cells, and ICC associated with intestinal motility, and intestinal LAB in loperamide-induced constipated rats. GOS consumption is associated with increased levels of *Bifidobacteria* and *Lactobacilli*, along with a group of intestinal microorganisms that selectively metabolize certain prebiotic/carbohydrate-type substances constantly because of resistant starch and prebiotic intake. In particular, GOS consumption seems to improve constipation by causing the proliferation of LAB in the intestinal flora, as well as increasing SCFA production.

## Supplementary Materials

The following are available online at https://www.mdpi.com/2075-4426/10/4/161/s1, Figure S1: Effects of galacto-oligosaccharide (GOS) administration on mRNA expression of IL-1 beta (A), IL-6 (B) and tumor necrosis factor-alpha (C) in loperamide-induced constipated rats, Figure S2: Average relative abundance of microbial communities at the level of phylum and genus in five-group (Nor, Con, PC, GL, and GH). Superscripts indicate (A) phylum and (B) genus.
