# Supplementary material for: Changes in the Gut Microbiome after Galacto-Oligosaccharide Administration in Loperamide-Induced Constipation
Source: J Pers Med. 2020 Oct 10;10(4):161. doi: 10.3390/jpm10040161 (PMC7711924; doi:10.3390/jpm10040161)
Supplement: Supplementary file 1 [file jpm-10-00161-s001.pdf]

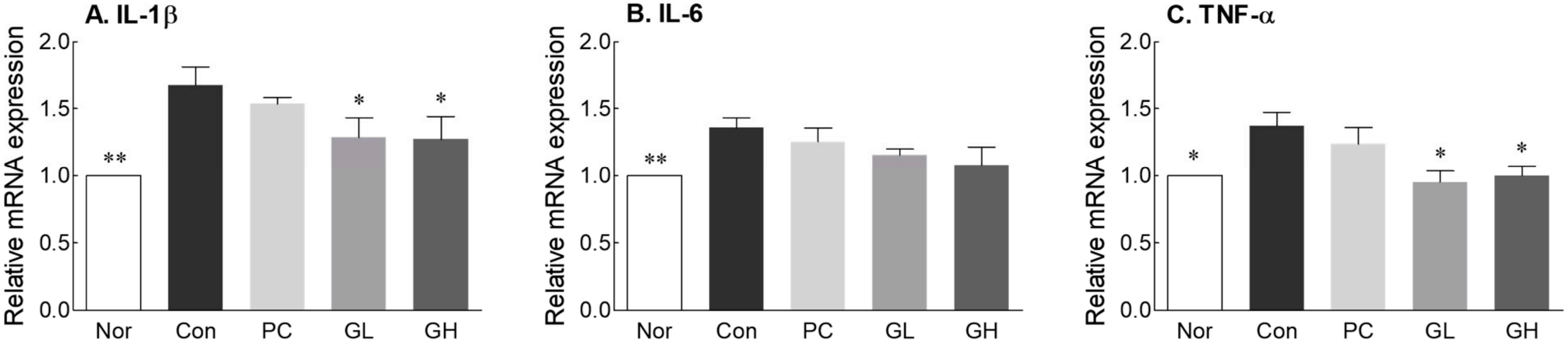

Sup. 1. Effects of galacto-oligosaccharide (GOS) administration on mRNA expression of IL-1 beta (A), IL-6 (B) and tumor necrosis factor-alpha (C) in loperamide-induced constipated rats. Data are represented as the mean ± standard error of the mean, and different symbols indicate significance at $^{*}p < 0.05$ and $^{**}p < 0.01$ vs. control group. Nor: normal group, Con: loperamide-treated group, PC: 70 mg/kg phenolphthalein-administrated group after loperamide-induced constipation, GL: low dose (100 mg/kg) of GOS-administrated group after loperamide-induced constipation, GH: high dose (200 mg/kg) of GOS-administrated group after loperamide-induced constipation.

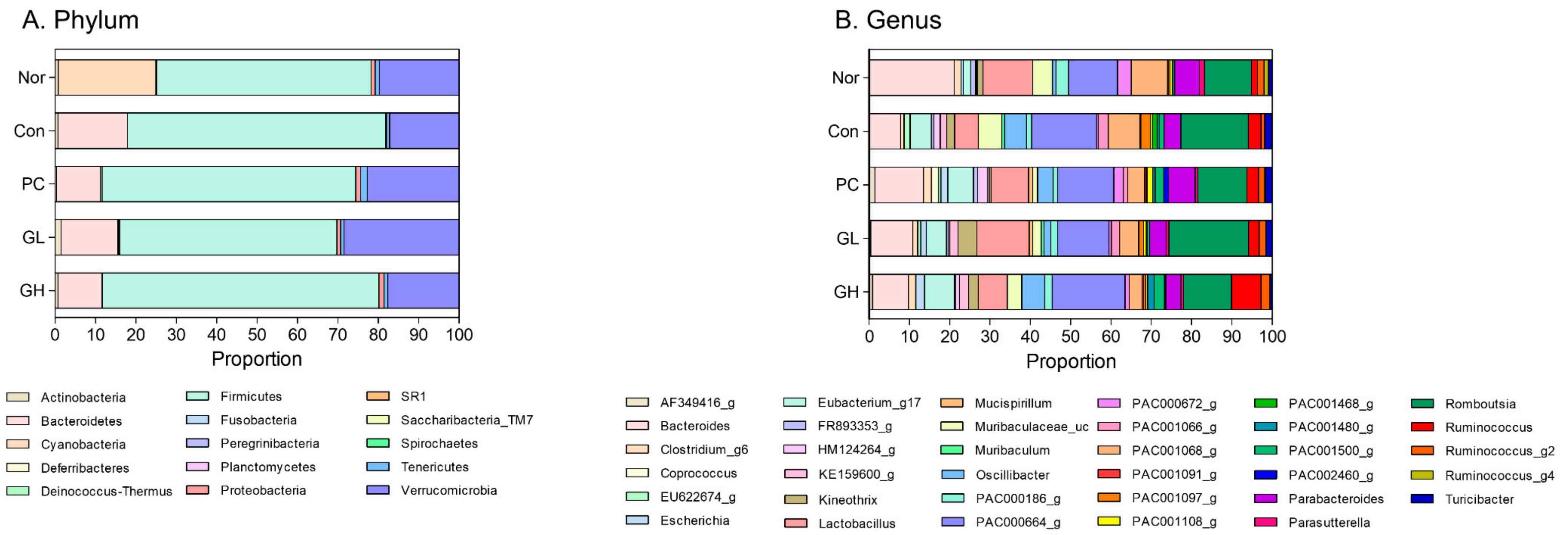

Sup. 2. Average relative abundance of microbial communities at the level of phylum and genus in five-group (Nor, Con, PC, GL, and GH). Superscripts indicate (A) phylum and (B) genus. Nor: normal group, Con: loperamide-treated group, PC: 70 mg/kg phenolphthalein-administrated group after loperamide-induced constipation, GL: low dose (100 kg/mg) of galacto-oligosaccharide (GOS)-administrated group after loperamide-induced constipation, GH: high dose (200 mg/kg) of GOS-administrated group after loperamide-induced constipation.
